# RAFT Synthesis and Characterization of Poly(Butyl-*co*-2-(*N*,*N*-Dimethylamino)Ethyl Acrylates)-*block*-Poly(Polyethylene Glycol Monomethyl Ether Acrylate) as a Photosensitizer Carrier for Photodynamic Therapy

**DOI:** 10.3390/ma16114192

**Published:** 2023-06-05

**Authors:** Makoto Obata, Shiho Hirohara

**Affiliations:** 1 Graduate Faculty of Interdisciplinary Research, University of Yamanashi, 4-4-37 Takeda, Kofu 400-8510, Japan; 2 Department of Chemical and Biological Engineering, National Institute of Technology (KOSEN), Ube College, 2-14-1 Tokiwadai, Ube 755-8555, Japan; hirohara@ube-k.ac.jp

**Keywords:** block copolymer, polymer micelle, photodynamic therapy

## Abstract

Polymer micelles are promising drug delivery systems for highly hydrophobic photosensitizers in photodynamic therapy (PDT) applications. We previously developed pH-responsive polymer micelles consisting of poly(styrene-*co*-2-(*N*,*N*-dimethylamino)ethyl acrylate)-*block*-poly(polyethylene glycol monomethyl ether acrylate) (P(St-*co*-DMAEA)-*b*-PPEGA) for zinc phthalocyanine (ZnPc) delivery. In this study, poly(butyl-*co*-2-(*N*,*N*-dimethylamino)ethyl acrylates)-*block*-poly(polyethylene glycol monomethyl ether acrylate) (P(BA-*co*-DMAEA)-*b*-PPEGA) was synthesized via reversible addition and fragmentation chain transfer (RAFT) polymerization to explore the role of neutral hydrophobic units in photosensitizer delivery. The composition of DMAEA units in P(BA-*co*-DMAEA) was adjusted to 0.46, which is comparable to that of P(St-*co*-DMAEA)-*b*-PPEGA. The size distribution of the P(BA-*co*-DMAEA)-*b*-PPEGA micelles changed when the pH decreased from 7.4 to 5.0, indicating their pH-responsive ability. The photosensitizers, 5,10,15,20-tetrakis(pentafluorophenyl)chlorin (TFPC), 5,10,15,20-tetrakis(pentafluorophenyl)porphyrin (TFPP), protoporphyrin IX (PPIX), and ZnPc were examined as payloads for the P(BA-*co*-DMAEA)-*b*-PPEGA micelles. The encapsulation efficiency depended on the nature of the photosensitizer. TFPC-loaded P(BA-*co*-DMAEA)-*b*-PPEGA micelles exhibited higher photocytotoxicity than free TFPC in the MNNG-induced mutant of the rat murine RGM-1 gastric epithelial cell line (RGK-1), indicating their superiority for photosensitizer delivery. ZnPc-loaded P(BA-*co*-DMAEA)-*b*-PPEGA micelles also exhibited superior photocytotoxicity compared to free ZnPc. However, their photocytotoxicity was lower than that of P(St-*co*-DMAEA)-*b*-PPEGA. Therefore, neutral hydrophobic units, as well as pH-responsive units, must be designed for the encapsulation of photosensitizers.

## 1. Introduction

Photodynamic therapy (PDT) is a promising cancer treatment that is minimally invasive, which allows for the maintenance of a high quality of life [1,2,3,4]. PDT photosensitizers must meet several requirements, such as non-toxicity in the dark, efficient reactive oxygen species (ROS) generation upon photoirradiation, absorption ability in the extended wavelength region, accumulation at tumor sites, and practical water solubility [5,6,7,8,9,10]. From the viewpoint of molecular design, these requirements often conflict with each other, especially light-absorbing ability and water solubility. Most PDT photosensitizers have a highly planar and rigid molecular structure that absorbs low-energy light. However, this structural feature tends to form aggregates, thereby reducing water solubility. Moreover, additional synthetic efforts must be devoted to gain practical water solubility, making PDT photosensitizers costly.

Drug delivery systems can provide alternatives for overcoming these conflicting demands. Among the various types of carriers, polymer micelles of amphiphilic block copolymers are a promising class of carriers because of their core–corona nanostructure that can hold hydrophobic molecules, their potential tumor-accumulating nature due to the enhanced permeation and retention (EPR) effect, and their cost-effective mass production owing to recently developed controlled radical polymerization. Various amphiphilic block copolymers, such as poly(ε-caprolactone)-*block*-poly(ethylene glycol) (PCL-*b*-PEG) [11,12,13,14,15,16,17,18,19], poly(l-lactide)-*block*-poly(ethylene glycol) (PLLA-*b*-PEG) [14], polystyrene-*block*-poly(ethylene glycol) (PSt-*b*-PEG) [20,21,22], and poly(ethylene glycol)-*block*-poly(propylene glycol)-*block*-poly(ethylene glycol) (PEG-*b*-PPO-*b*-PEG) [23,24,25,26,27], have been investigated for PDT and closely related cancer therapies, such as photothermal therapy (PTT). For example, Yan et al. prepared protoporphyrin IX (PPIX)-loaded PCL-*b*-PEG micelles and examined their photocytotoxicity in the human breast cancer cell line MDA-MB-231 [15]. They found that PPIX-loaded micelles exhibited excellent photocytotoxicity after erlotinib pretreatment. Gibot et al. investigated PCL-*b*-PEG and PSt-*b*-PEG micelles as pheophorbide *a* (Pheo) carriers [14]. Both Pheo-loaded micelles exhibited higher PDT efficacy than free pheophorbide *a* in the 2D monolayers and 3D spheroids of human HCT-116 colorectal cancer cells (CCL-247) and A375 melanoma cancer cells (CRL-1619). Recently, we developed novel amphiphilic block copolymers for PDT photosensitizer delivery [28,29,30,31]. Our polymers had two characteristic features: (1) a relatively short hydrophobic segment consisting of a hydrophobic monomer with pH-responsive units, 2-(*N*,*N*-dimethylamino)ethyl acrylate (DMAEA), and (2) a long brush-type hydrophilic segment consisting of polyethylene glycol monomethyl ether acrylate (PEGA) [28,29]. The significant size difference between the hydrophobic and hydrophilic segments resulted in a small packing parameter that prevented the formation of large aggregates. During cellular internalization, the pH-responsive units destabilize the aggregates in weakly acidic microenvironments, such as tumor sites and endosomes. According to this molecular design, poly(styrene-*co*-2-(*N*,*N*-dimethylamino)ethyl acrylate)-*block*-poly(polyethylene glycol monomethyl ether acrylate) (P(St-*co*-DMAEA)-*b*-PPEGA) was synthesized, and its performance was examined in the delivery of zinc phthalocyanine (ZnPc) as a PDT photosensitizer. ZnPc-loaded P(St-*co*-DMAEA)-*b*-PPEGA micelles exhibited superior photocytotoxicity compared to free ZnPc and ZnPc-loaded polymer micelles without pH-responsive units. Therefore, pH-responsive units are adequate as the carrier of the PDT photosensitizer. However, little is known about the role of simple hydrophobic units, such as styrene, in the photosensitizer carrier. In addition, polystyrene is suspected to be hazardous owing to the potential endocrine-disrupting nature of the oligomer [32]. Therefore, a pH-responsive amphiphilic block copolymer, poly(butyl-*co*-2-(*N*,*N*-dimethylamino)ethyl acrylates)-*block*-poly(polyethylene glycol monomethyl ether acrylate) (P(BA-*co*-DMAEA)-*b*-PPEGA), was synthesized via reversible addition and fragmentation chain transfer (RAFT) polymerization as an alternative photosensitizer carrier to examine the effect of hydrophobic units on the performance of the photosensitizer carrier. The pH-responsive nature, the photosensitizer-loading ability of the empty polymer micelles, and the photocytotoxicity of photosensitizer-loaded polymer micelles were examined.

## 2. Materials and Methods

### 2.1. Materials and Analytical Techniques

All the chemicals were of analytical grade. First, 1,4-Dioxane was purchased from FUJIFILM Wako Pure Chemical Corporation (Osaka, Japan) and distilled before use. Butyl acrylate (BA) and 2-(*N*,*N*-dimethylamino)ethyl acrylate (DMAEA) were purchased from Tokyo Chemical Industry Co., Ltd. (Tokyo, Japan) and purified by vacuum distillation. Polyethylene glycol methyl ether acrylate (PEGA; average molecular mass = 480) and 1,4-bis(trimethylsilyl)benzene (BTMSB) were purchased from Sigma-Aldrich (Tokyo, Japan). DMAEA and PEGA were passed through a short column filled with aluminum oxide immediately before use to remove impurities and inhibitors. Azobisisobutyronitrile (AIBN) was purchased from FUJIFILM Wako Pure Chemical Corporation and purified by recrystallization from methanol. Protoporphyrin IX (PPIX) was purchased from FUJIFILM Wako Pure Chemical Corporation. Zinc phthalocyanine (ZnPc) was purchased from Kanto Chemical Co., Inc. (Tokyo, Japan). *N*-(4-Trifluoromethylbenzyl)-4-cyano-4-(dodecylthiothiocarbonyl)thiopentanamide (CF_3_-CDSP), 5,10,15,20-tetrakis(pentafluorophenyl)porphyrin (TFPP), and 5,10,15,20-tetrakis(pentafluorophenyl)chlorin (TFPC) were prepared as previously reported [30,33,34]. Acetate-buffered saline ABS (pH 5) was prepared by dissolving sodium chloride (8.8 g, 150 mmol), potassium chloride (0.332 g, 4.45 mmol), acetic acid (0.183 g, 3.05 mmol), and sodium acetate (0.570 g, 6.95 mmol) in deionized water (1 L).

The ^1^H and ^19^F NMR spectra were measured using an AVANCE III HD spectrometer (500 MHz; Bruker Biospin K.K., Yokohama, Japan). The excitation spectra were measured using a spectrofluorometer (FP-6300, JASCO Co., Ltd. (Tokyo, Japan)). Preparative gel permeation chromatography (preparative GPC) was carried out using an LC-908 Recycling Preparative high-performance liquid chromatography system (Japan Analytical Industry Co., Tokyo, Japan) using two polystyrene gel columns (JAIGEL-2.5H and JAIGEL-2H) as the stationary phase and CHCl_3_ as the eluent. Size-exclusion chromatography (SEC) was performed using a high-performance liquid chromatography instrument (pump, LC-20AT; refractive index detector, RID-10A, Shimadzu Co.). Styragel HR4 (7.8 × 300 mm; Waters Co., Milford, MA, USA), Styragel HR3 (7.8 × 300 mm), and Styragel HR1 (7.8 × 300 mm) columns were used at 40 °C as the stationary phase. Tetrahydrofuran was used as the mobile phase at a flow rate of 1 mL min^−1^. The plots of log *M* vs. retention volume were prepared using 11 polystyrene standards (Showa Denko, K.K., Tokyo, Japan) ranging in molecular mass from 1.31 to 3740 kg mol^−1^. The number average molar mass (*M*_n_) and dispersity (*M*_w_/*M*_n_) were calculated using polystyrene calibration. The size distribution measurement was performed with a Zetasizer Nano ZSP (ZEN5600, Malvern Instruments, Herrenberg, Germany) equipped with a He−Ne laser (*λ* = 633 nm).

### 2.2. Synthesis and Characterization of P(BA-co-DMAEA) macroCTA

BA (765.3 mg, 6.0 mmol), DMAEA (567.7 mg, 4.0 mmol), CF_3_-CDSP (55.85 mg, 0.10 mmol), AIBN (1.63 mg, 0.010 mmol), and BTMSB (11.21 mg, 0.050 mmol) were placed in a test tube and completely dissolved. The test tube was tightly sealed with a rubber septum and cooled in an ice bath. Nitrogen was bubbled through the solution for 30 min. The test tube was then stirred at 60 °C for 3 h. An aliquot of the polymerization mixture was taken before and after polymerization to determine the monomer conversion via quantitative ^1^H NMR spectroscopy. The polymerization mixture was evaporated under reduced pressure to remove unreacted monomers. The crude product was purified using preparative GPC to obtain the P(BA-*co*-DMAEA) macromolecular chain transfer agent (macroCTA) as a yellow viscous product. The yield was 356.0 mg (25%) and the total monomer conversion was 0.21 (DP_n_ = 21, *F*_DMAEA_ = 0.46, *M*_n,NMR_ = 3397, *M*_n_ = 3230, and *M*_w_/*M*_n_ = 1.13 (polystyrene std.)).

### 2.3. Synthesis and Characterization of P(BA-co-DMAEA)-b-PPEGA

P(BA-*co*-DMAEA) macroCTA (33.4 mg, 0.0098 mmol), PEGA (481.5 mg, 1.0 mmol), AIBN (0.33 mg, 0.002 mmol), BTMSB (1.08 mg, 0.005 mmol), and 1,4-dioxane (1 mL) were placed in a test tube and thoroughly dissolved. The tube was tightly sealed with a rubber septum. Nitrogen was bubbled through the solution for 30 min. The test tube was then stirred at 60 °C for 24 h. An aliquot of the polymerization mixture was taken before and after polymerization to determine the monomer conversion via quantitative ^1^H NMR spectroscopy. The solution was poured into hexane and the supernatant was removed via decantation. The crude product was purified using preparative GPC to obtain P(BA-*co*-DMAEA)-*b*-PPEGA as a pale yellow viscous product. The yield was 349.3 mg (68%) and conversion of PEGA was 0.83 (DP_n,BA+DMAEA_ = 21, *F*_DMAEA_ = 0.46, DP_n,PEGA_ = 108, *M*_n,NMR_ = 55,237, *M*_n_ = 16,900, and *M*_w_/*M*_n_ = 1.28 (polystyrene std.)).

### 2.4. Preparation of Polymer Micelles in Buffered Saline

The typical procedure was as follows: P(BA-*co*-DMAEA)-*b*-PPEGA (12.8 mg) was dissolved in *N*,*N*-dimethylformamide (DMF; 1 mL). The solution was dialyzed with phosphate-buffered saline (PBS) using Spectra/Por^®^ Biotech cellulose ester dialysis membranes (molecular weight cut-off (MWCO): 500–1000 Da) for 3 days. The polymer concentration was adjusted to 2 mg mL^−1^ using PBS.

### 2.5. Preparation of Photosensitizer-Loaded Polymer Micelles in PBS

The typical procedure was as follows: P(BA-*co*-DMAEA)-*b*-PPEGA (15.0 mg) was dissolved in 121 μM TFPC in DMF solution (3 mL). The solution was dialyzed with PBS using Spectra/Por^®^ Biotech cellulose ester dialysis membranes (MWCO: 500–1000 Da) for 3 days. The polymer concentration was adjusted to 2 mg mL^−1^ using PBS. The photosensitizer concentration in the resulting polymer micelles was determined via UV-vis spectroscopy using a plate reader (Multiscan JX, Thermo Fisher Scientific Co., Yokohama, Japan) after dilution ten times with DMF. The initial concentrations of TFPC, TFPP, PPIX, and ZnPc in DMF solution were 121, 42.8, 111, and 72 μM, depending on the solubility of the photosensitizer in DMF.

### 2.6. Critical Micelle Concentration

Critical micelle concentration (CMC) was evaluated by fluorospectroscopy using pyrene as a microenvironment probe. The typical procedure was as follows: a stock solution of polymer micelles was diluted with distilled water in a concentration range of 1000–5.12 × 10^−4^ mg L^−1^. An aqueous pyrene solution (2 μM; 160 μL) containing 0.1 vol% acetone was added to the diluted polymer solution (160 μL) and then thoroughly mixed. The solution was maintained at 25 °C for 1 h in the dark. The fluorescence intensities at 390 nm were measured upon photoexcitation at 333 nm (*I*_333_) and 338 nm (*I*_338_) at 25 °C. The fluorescence intensity ratio (*I*_338_/*I*_333_) was plotted as a function of the logarithm of the polymer concentration. The CMC value was determined as the intersection point of the two tangents in the plot at higher and lower concentrations.

### 2.7. In Vitro Photocytotoxicity Test

#### 2.7.1. Cell Culture

An MNNG-induced mutant of the rat murine RGM-1 gastric carcinoma mucosal cell line (RGK-1) was kindly provided by Dr. Hirofumi Matsui (Faculty of Medicine, University of Tsukuba, Ibaraki, Japan). RGK-1 cells were grown in a 1:1 mixture of Dulbecco’s modified Eagle’s medium (DMEM) and F12 Ham’s medium (Sigma-Aldrich Japan, Tokyo, Japan) supplemented with 10 vol% fetal calf serum (FCS; Hyclone Laboratories, Inc., Logan, UT, USA) and 1 vol% Antibiotic-Antimycotic (Life Technologies, Tokyo, Japan).

#### 2.7.2. Sample Preparation

- Photosensitizer-loaded polymer micelles: A PBS solution of polymer micelles was diluted with PBS to double the predetermined concentration, and then completely mixed with the same volume of the culture medium.
- Bare TFPC: To avoid precipitation, a DMSO solution of TFPC was added to the culture medium to obtain a culture medium containing twice the target concentration of ZnPc and 2 vol% DMSO. The culture medium was completely mixed with the same volume of PBS.

#### 2.7.3. Photocytotoxicity

The cytotoxicity in RGK-1 cells was tested as follows: RGK-1 cells (5 × 10^3^ cells/well) in 100 μL of culture medium (100 μL) were plated in a 96-well plate (Thermo Fisher Scientific Co., Yokohama, Japan). The plates were then incubated for 24 h (37 °C, 5% CO_2_). After removing the culture medium, 100 μL sample solutions were added to each well. The plates were then co-incubated with each sample for 24 h. After washing the cells twice with PBS, 100 μL of the culture medium was added to each well. The cells were irradiated with light from a 100 W halogen lamp (KBEX-102A, Ushio Inc., Tokyo, Japan) equipped with a Y-50 cut-off filter (*λ* > 500 nm, Toshiba Corporation, Tokyo, Japan) and a water jacket. UV-vis power meter (ORION/TH, Ophir Optronics Ltd., Jerusalem, Israel) was used to determine the light intensity. The photoirradiation time was regulated to acquire the predetermined light doses of 0 (dark) and 20 J cm^−2^. WST-8^®^ reagent (8 μL) from Cell Counting Kit-8 (Dojindo Laboratories, Kumamoto, Japan) was applied to each well at 24 h after photoirradiation to determine the mitochondrial activity of NADH dehydrogenase in the cells according to the manufacturer’s instructions. The absorbance at 450 nm was determined using a plate reader (Multiscan JX, Thermo Fisher Scientific Co., Yokohama, Japan). The percentage of cell survival was calculated by the six replicate experiments using the value for photo-irradiated cells without photosensitizers under the same conditions as the reference. All values of the cell-survival rate were expressed as mean ± standard deviation [30].

## 3. Results and Discussion

### 3.1. Synthesis of P(BA-co-DMAEA)-b-PPEGA

**Scheme 1:**
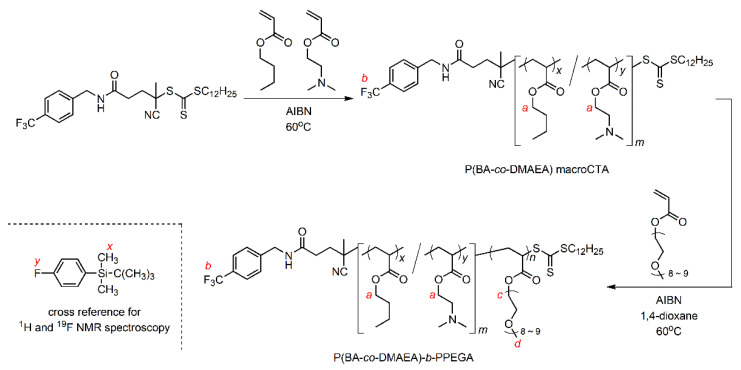
Synthesis of P(BA-*co*-DMAEA)-*b*-PPEGA via RAFT polymerization. The red letters are symbols for peak assignments in Figure 2.

**Figure 1:**
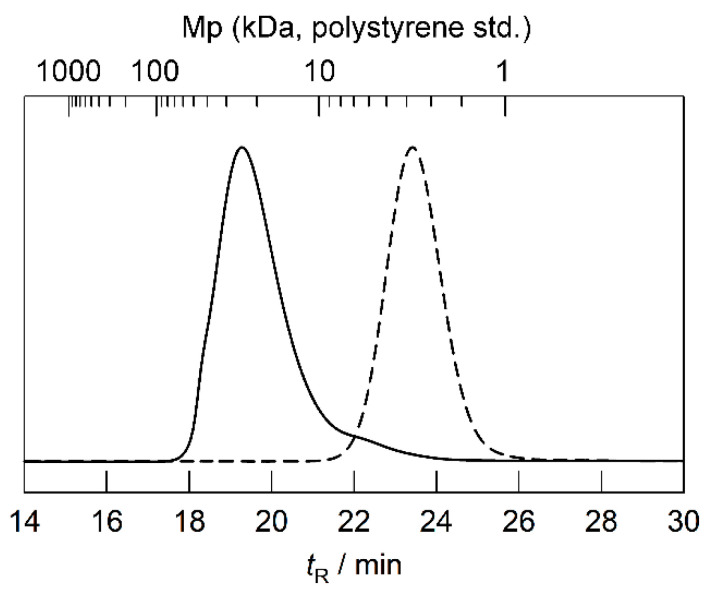
SEC traces of P(BA-*co*-DMAEA) macroCTA (broken line) and P(BA-*co*-DMAEA)-*b*-PPEGA (solid line).

**Figure 2:**
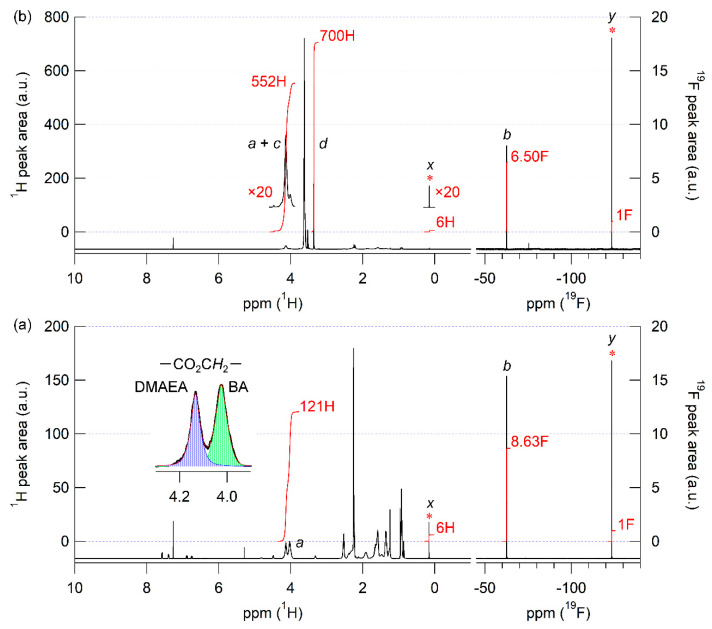
^1^H (right panel) and ^19^F NMR spectra (right panel) of P(BA-*co*-DMAEA) macroCTA (**a**) and P(BA-*co*-DMAEA)-*b*-PPEGA (**b**) with 4-fluorophenoxy-*tert*-butyldimethylsilane as a cross-reference in CDCl_3_. The peak assignments a, b, c, d, x, and y are presented in Scheme 1. Asterisks indicates the peaks attributable to the cross-reference. The inset of (**a**) is the expanded ^1^H NMR spectra ranged from 3.8 to 4.3 with the best-fitted curves.

The RAFT copolymerization of BA with DMAEA was performed using CF_3_-CDSP and AIBN as a chain transfer agent and an initiator, respectively, at 60 °C for 3 h (Scheme 1). The total monomer conversion was determined to be 0.21 via quantitative ^1^H NMR spectroscopy. Unfortunately, it is difficult to evaluate the conversions of each monomer separately. The *M*_n_ and *M*_w_/*M*_n_ values were evaluated to be 3230 and 1.13, respectively, via SEC calibrated using a polystyrene standard (Figure 1). The *M*_w_/*M*_n_ value was sufficiently low, which is typical of RAFT polymerization. Figure 2a shows the ^1^H and ^19^F NMR spectra of the resulting polymer in the presence of 4-fluorophenoxy-*tert*-butyldiemthylsilane as a cross-reference for normalization of peak areas between these two spectra. The mole fraction of DMAEA (*F*_DMAEA_) was determined to be 0.46 based on the peak area ratio in the methylene region from 3.8 to 4.3 ppm. The trifluoromethyl group at the initiating end was unambiguously identified in the ^19^F NMR spectrum by virtue of the nuclear specificity of NMR spectroscopy. The molar ratio between the polymer and the cross-reference was determined using ^19^F NMR spectroscopy, while the molar ratio between the BA and DMAEA units to the cross-reference was determined using ^1^H NMR spectroscopy. The degree of polymerization, DP_n_, was calculated to be 21 by combining the two ratios. The chain extension of P(BA-*co*-DMAEA) macroCTA with PEGA was performed using AIBN as an initiator in 1,4-dioxane at 60 °C. Figure 1 shows SEC traces of the resulting polymers. The SEC trace shifted toward a shorter retention time compared to that of P(BA-*co*-DMAEA) macroCTA, indicating successful chain extension. Figure 2b shows the ^1^H and ^19^F NMR spectra of the resulting polymer in the presence of 4-fluorophenoxy-*tert*-butyldimethylsilane as a cross-reference. The initiating and terminating end groups were substantially undetectable in the ^1^H NMR spectrum because of the extremely intense peak at 3.6 ppm attributable to the PPEGA segment. However, the ^19^F NMR spectrum shows a clear peak attributable to the trifluoromethyl groups at the initiating end. By combining quantitative information from the ^1^H and ^19^F NMR spectra, the DP_n_ value of the PPEGA segments was calculated to be 108. Hence, this technique, which uses a cross-reference for ^1^H and ^19^F NMR spectroscopy, is immensely powerful for quantitative end-group analysis.

### 3.2. Preparation and Characterization of Polymer Micelles

**Figure 3:**
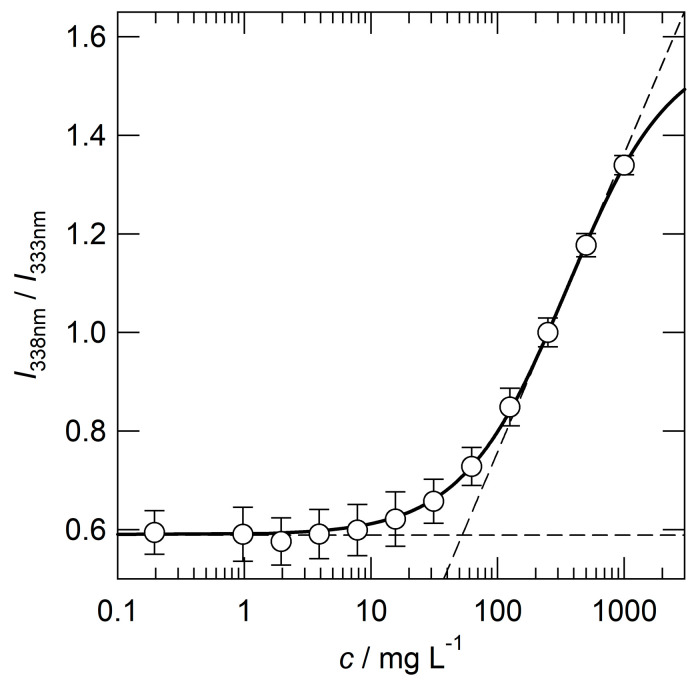
*I*_338_/*I*_333_ values as a function of the concentration of P(BA-*co*-DMAEA)-*b*-PPEGA in PBS. The concentration of pyrene was 1 μM.

**Figure 4:**
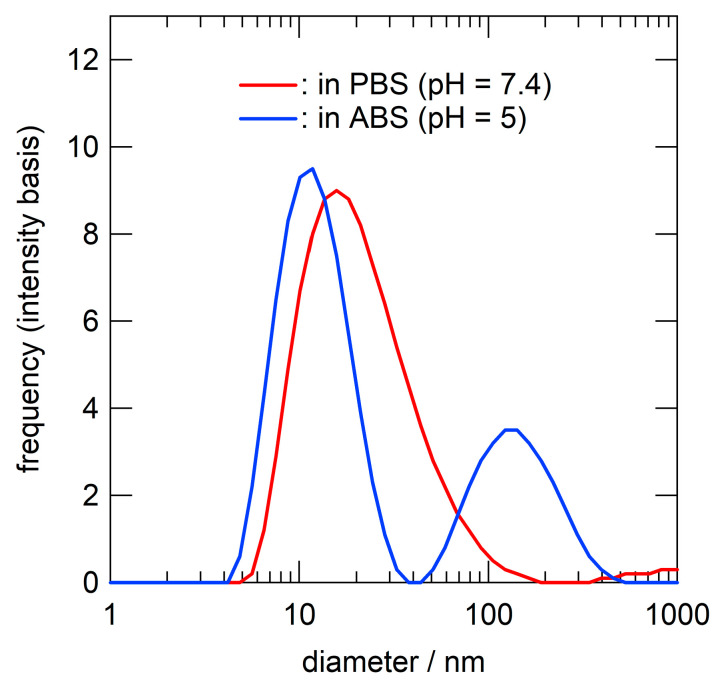
Size distributions of P(BA-*co*-DMAEA)-*b*-PPEGA micelle in PBS (red, pH = 7.4) and ABS (blue, pH = 5). The polymer concentration was 2000 mg L^−1^.

Polymer micelles were prepared via dialysis. Briefly, a DMF solution of P(BA-*co*-DMAEA)-*b*-PPEGA was dialyzed against phosphate-buffered saline (PBS) to obtain a clear, colorless solution. The polymer concentration was adjusted to 2000 mg L^−1^ with PBS. The critical micelle concentration (CMC) was evaluated via fluorophotometry using pyrene as a hydrophobic probe. Figure 3 shows the plots of the fluorescence intensity ratio (*I*_338_/*I*_333_) as a function of the logarithm of the polymer concentration. The crossing point of the two tangent lines gives a CMC value of 53 mg L^−1^. This value is comparable to that of P(St-*co*-DMAEA)-*b*-PPEGA (40 mg L^−1^) [29]. The size distributions of the polymer micelles were evaluated by dynamic light scattering (DLS) measurement, as shown in Figure 4. The hydrodynamic diameter, *D*_h_, was estimated to be 18 nm. This value was comparable to that of P(St-*co*-DMAEA)-*b*-PPEGA [29]. The pH-responsive nature of the micelles was examined via DLS using polymer micelles prepared with ABS (pH 5) instead of PBS (pH 7.4). The blue line in Figure 4 shows the apparent size distribution of the polymer micelles in ABS. This significant change suggests the pH-responsive nature of the polymer micelles. It should be noted that the inverse Laplace transform of the autocorrelation function of the sample in ABS (blue line) is not so reliable for unstable aggregates. Similar results was reported by Bovone et al. in their DLS study on polymer micelles during solvent quality change [35].

### 3.3. In Vitro Photocytotoxicity of Photosensitizer-Loaded Polymer Micelles

**Scheme 2:**
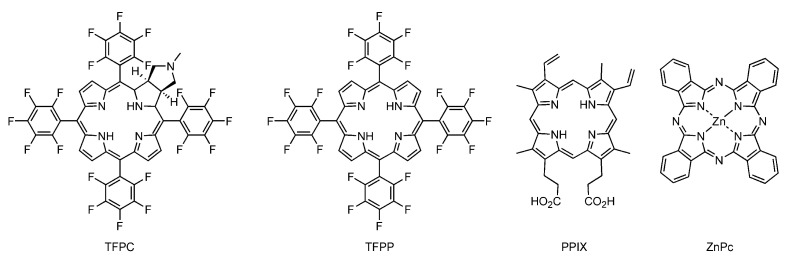
Structures of photosensitizers.

Photosensitizer-loaded polymer micelles were prepared via dialysis using a photosensitizer-containing DMF solution instead of pure DMF. After removing unloaded photosensitizer precipitates via filtration with a membrane filter (pore size was 0.5 μm), the photosensitizer concentration was determined photometrically in DMF solution (Table 1). TFPC, TFPP, PPIX, and ZnPc were used as photosensitizers (Scheme 2). The encapsulation efficiency (E.E.), which is the fraction of photosensitizer loaded to the photosensitizer in the feed, varied from 57 to 100%. The loading capacity (L.C.), the weight fraction of the loaded photosensitizer to all of the polymer micelles, varied from 0.49 to 2.4 wt%. Highly symmetric photosensitizers, such as TFPP and ZnPc, showed lower E.E. values, even with a low concentration of the photosensitizer in the feed, because these photosensitizers quickly precipitated out during the solvent quality change. In contrast to the *D*_4h_ symmetry of TFPP and ZnPc, the structures of TFPC (*C*_s_ symmetry) and PPIX (dissymmetry) were less symmetrical. In general, highly symmetric molecules exhibit lower solubility than highly dissymmetric molecules [36]. Hence, the less symmetric structures of TFPC and PPIX were advantageous to prohibit the precipitation out during encapsulation. In addition, the carboxylic groups of PPIX might be helpful for encapsulation by interacting with DMAEA units.

**Figure 5:**
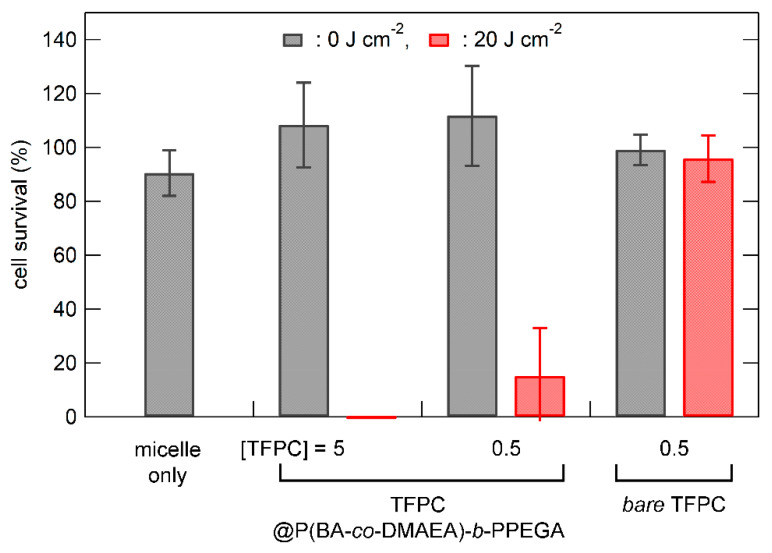
Dark and photocytotoxicity of the P(BA-*co*-DMAEA)-*b*-PPEGA micelle (denoted as micelle only; copolymer = 1000 mg L^−1^), TFPC-loaded P(BA-*co*-DMAEA)-*b*-PPEGA micelle, and bare TFPC in RGK-1 cells as a function of the light dose. The photoirradiation was provided by a 100 W halogen lamp equipped with a Y-50 cutoff filter (*λ* > 500 nm). The values are the mean ± standard deviation of six replicate experiments.

**Figure 6:**
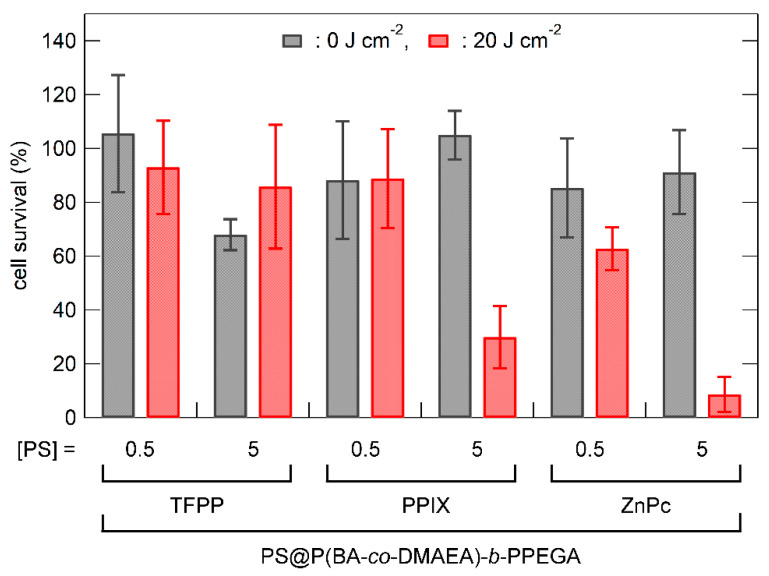
Dark and photocytotoxicity of the photosensitizer(PS)-loaded P(BA-*co*-DMAEA)-*b*-PPEGA micelle and bare TFPC in RGK-1 cells as a function of the light dose. The photoirradiation was provided by a 100 W halogen lamp equipped with a Y-50 cutoff filter (*λ* > 500 nm). The values are the mean ± standard deviation of six replicate experiments.

The photocytotoxicity of the photosensitizer-loaded polymer micelles was examined in RGK-1 cells. Empty polymer micelles showed no cytotoxicity at a polymer concentration of 1000 mg L^−1^ (Figure 5; micelles only). Figure 5 shows the dark and photocytotoxic effects of the TFPC-loaded polymer micelles and bare TFPC on RGK-1 cells. No cytotoxicity was observed without photoirradiation. Bare TFPC (without encapsulation in polymer micelles) showed no cytotoxicity with photoirradiation (*λ* > 500 nm; light dose, 20 J cm^−2^). In contrast, TFPC-loaded polymer micelles exhibited significant photocytotoxicity under the same TFPC concentration and photoirradiation conditions. Hence, P(BA-*co*-DAMEA)-*b*-PPEGA micelles act not only to solubilize hydrophobic photosensitizers but also to enhance photodynamic action. In order to elevate the PDT efficacy, the photosensitizers must have (1) efficient internalization into the cell, (2) no concentration quenching, and (3) localization in the vicinity of the target intracellular organelle or biomacromolecules. The encapsulation in polymer micelles is thought to enhance cellular internalization via the endocytotic pathway and to prevent the precipitation of photosensitizers and local concentration quenching. Figure 6 shows the photocytotoxicity of various photosensitizer-loaded polymer micelles in RGK-1 cells. In the case of TFPP, no significant photocytotoxicity was observed because of its high aggregation tendency and poor absorbing ability at wavelengths over 500 nm. PPIX-loaded polymer micelles exhibited detectable photocytotoxicity at the concentration of 5 μM with photoirradiation of 20 J cm^−2^. ZnPc-loaded polymer micelles showed higher photocytotoxicity than PPIX-loaded micelles, plausibly because of their higher absorption ability in extended wavelength regions. The PDT efficacy of ZnPc-loaded P(BA-*co*-DMAEA)-*b*-PPEGA micelles (approximately 60% survival ratio, 0.5 μM with 20 J cm^−2^ photoirradiation) was lower than that of P(St-*co*-DMAEA)-*b*-PPEGA (almost executed at 0.05 μM with 20 J cm^−2^ photoirradiation) [29]. These results suggest that the pH-responsive unit DMAEA, which acts as a PDT efficacy enhancing element, is not the only key factor in photosensitizer carriers. The pH-insensitive units, such as BA and St, also affect the PDT action through other factors, such as photosensitizer-solubilizing ability and significant incompatibility with the hydrophilic PPEGA segment to stabilize the core−corona microstructure.

## 4. Conclusions

A pH-responsive amphiphilic block copolymer, P(BA-*co*-DMAEA)-*b*-PPEGA, was successfully synthesized via successive RAFT polymerizations and fully characterized using ^1^H and ^19^F NMR spectroscopy and SEC. P(BA-*co*-DMAEA)-*b*-PPEGA afforded polymer micelles a hydrodynamic diameter of 18 nm via solvent change during dialysis. The pH-responsive nature of the polymer micelles was confirmed using DLS measurements in PBS and ABS. The TFPC-loaded polymer micelles exhibited higher photocytotoxicity than bare TFPC, verifying the effectiveness of the polymer micelles as photosensitizer carriers. The loading ability of the resulting polymer micelles depended on the nature of the photosensitizer. The ZnPc-loaded polymer micelles showed lower PDT efficacy than similar polymer micelles containing St units. Furthermore, in addition to pH-responsive units, pH-nonresponsive hydrophobic units must be suitably designed for PDT photosensitizers.

## Figures and Tables

**Table 1:** Preparation of photosensitizer-loaded P(BA-*co*-DMAEA)-*b*-PPEGA micelles.

Photosensitizer	*c*_PS_ (μM) *^a^*	E.E. (%) *^b^*	L.C. (%) *^c^*	*c*_PS_/*c*_polymer_*^d^*
TFPC	46	87	2.4	1.3
TFPP	10	57	0.49	0.28
PPIX	46	100	1.3	1.3
ZnPc	20	68	0.58	0.55

*^a^* The concentration of photosensitizer determined by UV-vis spectrophotometry. *^b^* The encapsulation efficiency, calculated as follows: (weight of the loaded photosensitizer in polymer micelles)/(weight of the photosensitizer in the feed) × 100. *^c^* The loading capacity, calculated as follows: (weight of the loaded photosensitizer in polymer micelles)/(weight of polymer micelles) × 100. *^d^* The molar ratio of photosensitizer to polymer.

## Data Availability

Not applicable.
